# The relationship between the Leicester cough questionnaire, eosinophilic airway inflammation and asthma patient related outcomes in severe adult asthma

**DOI:** 10.1186/s12931-017-0520-2

**Published:** 2017-03-04

**Authors:** Sushiladevi Natarajan, Robert C. Free, Peter Bradding, Lorcan McGarvey, Salman Siddiqui

**Affiliations:** 1 Department of Infection, Immunity and Inflammation, NIHR Respiratory Biomedical Research Unit, Glenfield Hospital, University of Leicester, Leicester, UK; 2 Centre for Infection & Immunity, School of Medicine, Dentistry and Biomedical Sciences, Queen’s University Belfast, Belfast, Ireland

**Keywords:** Asthma, Cough

## Abstract

**Background:**

Severe asthma is characterised by a variety of symptoms, which include chronic cough, however the mechanisms responsible for cough reflex hypersensitivity in asthma remain poorly elucidated. Current asthma patient-related outcome instruments such as the six-point Juniper Asthma Control Score (ACQ-6) and Asthma Quality of Life Questionnaire (AQLQ) were not primarily designed to capture cough and its related morbidity in asthma. The Leicester Cough Questionnaire (LCQ) is a patient-related outcome instrument designed to capture the health-related quality of life associated with cough. To date the LCQ has not been evaluated in a severe asthma population.

**Methods:**

We evaluated 262 extensively characterised adult patients with severe asthma attending the Leicester Severe Asthma Service. All patients had a clinician diagnosis of asthma and objective physiological evidence and met the ATS/ERS criterion for servere asthma. In all patients we evaluated a) the LCQ distribution and b) the relationships between the LCQ and ACQ-6, AQLQ, airway inflammation in sputum.

**Results:**

The LCQ demonstrated the following properties; mean: 15.0, standard deviation: 4.54, median: 15.48, and range: 11.6–19.2. We found a moderate correlation between LCQ and ACQ-6 (*r* = − 0.605, *p* < 0.0001) and a LCQ and AQLQ (*r* = 0.710, *p* < 0.0001). There was no relationship between LCQ and log_10_ sputum percentage eosinophils (%).

**Conclusion:**

A proportion of patients with severe asthma have a significant degree of cough-related morbidity that appears independent of eosinophilic airway inflammation and is not captured fully by existing asthma patient-reported outcome instruments. Our preliminary findings suggest that further research is now required to validate the LCQ and its responsiveness in severe asthma populations to capture cough-related morbidity and response to specific interventions.

## Introduction

Severe asthma is characterised by suboptimal asthma control in patients receiving step 4/5 therapy [1] and is associated with high levels of health care utilisation [2]. Severe asthma is associated with a range of co morbidities [3] (such as nasal polyps, suboptimal treatment adherence, as well as treatment refractory and difficult asthma) [1, 4].

Chronic cough is associated with physical and psychological morbidity [5], however the prevalence of chronic cough within severe asthma populations has not been determined, in part due to the lack of well-validated assessment tools.

Cough reflex hypersensitivity syndrome [6] may underlie the aetiology of chronic cough but the mechanisms are poorly understood and therapeutic options are limited. Similarly, the impact of chronic cough on the health status of patients with severe asthma is not well understood as existing asthma patient-related outcome measures such as the six-point Juniper Asthma Control Questionnaire (ACQ-6) and Asthma Quality of Life Questionnaire (AQLQ) were not primarily designed to capture cough and its related morbidity. There are a number of tools that can be used to understand and phenotype cough which include digital cough monitors [7], cough specific health status questionnaires such as the Leicester Cough Questionnaire (LCQ) and cough visual analogue scales [8, 9]. The LCQ is a patient-reported questionnaire designed to capture the health-related quality of life of cough and is responsive to changes in chronic cough [10]. More recently the LCQ has been used in asthma and chronic obstructive pulmonary disease (COPD) patients to assess the impact of gastro-oesophageal reflux disease (GORD) on cough-related morbidity [11] and has also been validated against the SF-12 questionnaire in COPD [12]. However the LCQ has not been evaluated in large carefully characterised severe asthma populations to date.

We sought to determine the spectrum of cough related morbidity in a severe asthma population, by measuring total LCQ and its sub domains and evaluating its relationship with standard asthma related questionnaires (ACQ-6, AQLQ) and percentage sputum eosinophils (a marker of the degree of eosinophilc bronchitis –a reported driver of cough in asthma).

## Methods

We evaluated 312 adult patients referred to the Leicester Difficult Asthma Service, using data from a local clinical management database. The database included all referral from primary and secondary care.

The use of data extracted from the local database for the purposes of research was approved by the Leicestershire, Northamptonshire, & Rutland Research Ethics Committee (REC Reference.13/EM/0287) and all patients gave their written informed consent.

The Leicester difficult asthma service, is a tertiary referral service for difficult asthma patients in the UK and receives both local and national referrals to evaluate patients with difficult to control asthma. The evaluation includes (i) confirmation of the diagnosis of asthma in all patients using objective measurements, including extensive phenotyping assessments (ii) systematic evaluation and treatment of comorbidities e.g., rhinosinusitis, non-adherence and (iii) evaluation for biological agents and bronchial thermoplasty after (i) and (ii).

All patients (*n* = 312) had an expert clinician diagnosis of asthma and one or more of the following objective physiological criteria; (i) positive methacholine PC_20_ ≤ 8 mg/ml, (ii) bronchodilator responsiveness of 200 ml and 12% following the administration of 200 μg of inhaled salbutamol or (iii) peak flow variation over a two week period of ≥20% [1].

22/312 patients were excluded from analyses as they did not have difficult to treat asthma and had milder disease (GINA treatment steps 1–3).

262/290 patients met the criteria met the American Thoracic Society/European Respiratory Society consensus criteria for severe asthma [13]. Therefore our final population for analysis consisted entirely of patients with severe asthma, defined according to international guidelines, all of whom had objective evidence of asthma and were being assessed and managed in a tertiary severe asthma centre in the UK.

Phenotyping assessments were performed in the stable state and in patients that had been free from exacerbations for ≥6 weeks. Patients underwent lung function measurements, including spirometry [post bronchodilator (400 μg salbutamol via a metered dose inhaler and spacer)], induced sputum samples for differential cell count and completed the LCQ, ACQ-6 and AQLQ questionnaires at a single clinical visit. Due to the association of cough with reflux and upper airway disease, self-reported gastrointestinal reflux, defined as the presence of heartburn, cough, nausea, chest pain or dysphagia after meals was documented, whilst the presence or absence of seasonal/perennial rhinitis and nasal polyposis was also recorded.

The LCQ is a nineteen-point questionnaire capturing three domains; Physical (8 items), Psychological (7 items) and Social (4 items). Each item has a score range 1 to 7. Item scores for each domain are summed and divided by the total number of items in each domain. The total LCQ is the sum of all domain scores. The minimum and maximum achievable LCQ total scores are 3 and 21 respectively [10]. A lower LCQ score signifies more cough. The LCQ Minimal clinically important difference (MCID) is 1.3 in chronic cough [14]. The Global Lung function Initiative (GLI) reference equations were used to calculate FEV_1_ Z score. Patients with abnormal spirometry had an FEV_1_ standardised residual of ≤ −1.64.

### Statistical analysis

Statistical analysis was performed using SPSS 22 (IBM Corporation, Somers, NY, USA) and Prism 6 (GraphPad Software Inc., La Jolla, California, USA). Data were analysed using Student’s *t*-test or Mann-Whitney *U* test for parametric and non-parametric data respectively and chi squared test for proportions. The threshold for statistical significance was set at *p* < 0.05. Correlations between continuous variables were calculated using Pearson’s correlation coefficient.

## Results

The clinical characteristics of the asthma population are shown in Table 1. The population reported here was similar to other UK severe asthma populations [15]. We did not systematically exclude smokers (see Table 1), however only 3/42 patients with a smoking history ≥ 10 pack years had spirometrically confirmed fixed airflow obstruction (compatible with ACOS).

**Table 1:** Clinical Characteristics of Severe Asthma Populationᅟ

	Asthma population (*n* = 262)
Age (years)	54.03 (14.9)
Sex (% male)	37.4
GINA Treatment Step^a^	5 (4–5) Step 4: 124 Step 5: 96
Sputum Eosinophil (%)^a^	3.5 (0.5-18.75)
% on ICS/LABA combination therapy (number)	94.5 (*n* = 247)
% on Montelukast (number)	27.1 (*n* = 71)
% on oral theophylline (number)	32.4 (*n* = 85)
% on Inhaled Tiotrpoium Bromide (number)	26.0 (*n* = 68)
% on maintenance oral prednisolone (number)	53.8 (*n* = 156)
Dose (mg/24 h) in those on Prednisolone	10 (5.0–12.5)^a^
Self-reported gastrointestinal reflux (%)	49.0
Nasal polyps (%) (number)	26.3 (*n* = 69)
Perennial/seasonal rhinitis % (number)	58.3 (*n* = 153)
Smoking history (pack years)	7.2 (2.6–20)
Exacerbations in past year	3 (1.0–6.0)^a^
ITU admissions in past year	0.0 (0.0–0.0)^a^
Total ITU admissions	0.0 (0.0–1.0)^a^
Hospital admissions in past year	0.0 (0.0–1.0)^a^
Unscheduled GP/A&E visits	3.0 (0.0–7.0)^a^
Smoking history (pack years)	7.2 (2.6–20)^a^
Proportion of current: ex smokers	Current smokers: *n* = 19 Ex-smokers: *n* = 76
% Smoking >10 pack years (number)	16 .0 (*n* = 42)
Post Bronchodilator FEV_1_ (% Predicted)	73.33 (35.7)
Post Bronchodilator FVC (% Predicted)	96.6 (154..4)
Post Bronchodilator FEV_1_/FVC (%)	66.82 (14.6)
GLI FEV_1_ Z score	−1.67 (2.68)
Bronchodilator reversibility (%)	14.13 (19.01)
ACQ-6 score^a^	2.50 (1.5–3.5)^a^
AQLQ score^a^	3.83 (3.0–5.1)^a^
LCQ Total score^a^	15.48 (11.6–19.2)^a^
LCQ Total score (females)^a^	14.9 (10.8–18.7) [*n* = 164]
LCQ Total Score (males)^a^	17.1 (12.8–19.8) [*n* = 98]
LCQ Total score (smoker <10 pack/years)	16.22 (11.3–19.0)
LCQ Total score (smoker ≥10 pack/years)	15.4 (12.7–19.8)
LCQ Physical^a^	4.75 (3.6–6.0)^a^
LCQ Psychological^a^	5.57 (4.0–6.7)^a^
LCQ Social^a^	5.38 (3.8–6.8)^a^

*GINA* Global Initiative for Asthma, *ICS/LABA* inhaled corticosteroids/long acting beta-2 agonist combination therapy, *ITU* Intensive care unit, *GP* General practice, *A&E* Accident and emergency, *FEV*_*1*_ Forced Expiratory Volume in one second, *FVC* Forced Vital Capacity, *GLI* Global Lung Function Initiative, *ACQ-6* Six-point Asthma Control Questionnaire, *AQLQ* Asthma Quality of Life Questionnaire, *LCQ* Leicester Cough Questionnaire. Data expressed as mean (standard deviation) or ^a^median (interquartile range)

**Fig. 1:**
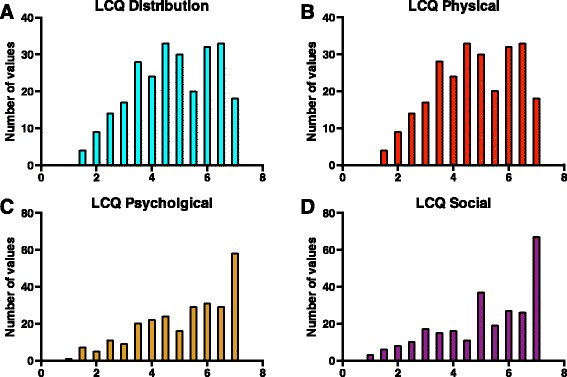
Distribution of the Leicester Cough Questionnaire. LCQ = Leicester Cough Questionnaire. Panel **a**: Histogram of the distribution of the Leicester cough questionnaire, Panel **b**-**d**: Histogram of the distribution of Leicester cough questionnaire domains

Figure 1a-d, shows the LCQ total and domain distributions. The LCQ demonstrated the following properties: a) Mean: 15.0, b) Standard deviation: 4.54, c) Median: 15.48, c) IQR: 11.6–19.2, d) Range: 4.0–21, e) 10^th^ percentile point: 8.20 and f) 90^th^ percentile point: 20.38.

**Fig. 2:**
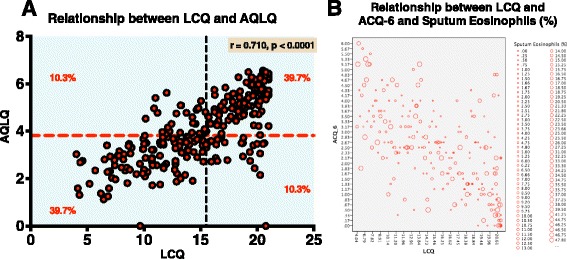
The relationships between the Leicester Cough Questionnaire and Eosinophilic airway Inflammation in sputum and Asthma Patient-Related Outcomes in Severe Asthma. LCQ = Leicester Cough Questionnaire, ACQ-6 = Six-point Asthma Control Questionnaire, AQLQ = Asthma Quality of Life Questionnaire Panel **a**: Correlation between the Leicester cough questionnaire and Asthma quality of life questionnaire. Pearson correlation coefficient: *r* = 0.710, *p* < 0.0001. *Dashed line* represents median. Percentage of subjects in each quadrant is shown. Panel **b**: Bubble plot representing correlations between the Leicester cough questionnaire and sputum eosinophils (%) (Pearson correlation coefficient: *r* = −0.106, *p* = 0.106) and Six-point Asthma control questionnaire (Pearson correlation coefficient: *r* = .0.605, *p* < 0.0001

There was a moderate negative correlation between LCQ and ACQ-6 (*r* = − 0.605, *p* < 0.0001) (see Fig. 2b), and a moderate positive correlation between LCQ and AQLQ (*r* = 0.710, *p* < 0.0001) (Fig. 2a). There was no correlation between LCQ and log_10_ sputum eosinophils (%) (*r* = −0.106, *p* = 0.106) or sputum neutrophils (data not shown). Similar and statistically significant correlations existed between LCQ and ACQ-6/AQLQ in GINA 4 and GINA 5 severe asthma populations respectively (data not shown).

The female asthma population (62.6%) had an LCQ of 14.9 (10.8–18.7), significantly lower LCQ when compared to males 17.1 (12.8–19.8), *p* = 0.0055.

Patients with asthma with self-reported gastrointestinal reflux (47.3%) were more likely to be female (67.7%, *p* < 0.0001) and had a significantly lower LCQ compared to patients without self-reported gastrointestinal reflux [median (IQR): 14.3 (10.8–17.8) vs.16.5 (12.8–19.7), *p* = 0.0015]. However there was no significant difference in LCQ between female patients with asthma with self-reported gastrointestinal reflux (32.0%) and without self-reported gastrointestinal reflux (30.5%) [median (IQR): 14.31 (10.9–16.9) vs. 15.83 (10.2–19.5), *p* = 0.1835].

There was no significant difference in LCQ between patients with asthma with nasal polyps and patients with asthma without nasal polyps [median (IQR): 14.7 (10.7–19.7) vs. 15.5 (11.9–18.8), *p* = 0.7906]. There was also no significant difference in LCQ between patients with asthma with perennial/seasonal rhinitis compared to patients with asthma without perennial/seasonal rhinitis [median (IQR): 15.21 (11.6–19.0) vs. 16.36 (15.7–20.0), *p* = 0.3680).

There was no significant difference in the total LCQ score between smokers with ≥10 pack/years and smokers with <10 pack/years (median (IQR): 15.4 (12.7–19.8) vs.16.22 (11.3–19.0), *p* = 0.5755).

## Discussion

We have chosen the LCQ, a simple and objective measure of cough to evaluate the burden of cough in severe asthma and its relationships to existing asthma patient-related outcome measures and eosinophilic airway inflammations.

We identified that a significant proportion of patients with severe asthma demonstrated high levels of cough-related morbidity as evidenced by the LCQ. The 10^th^ percentile LCQ (8.20) and median LCQ (15.48) in severe asthma when compared to other chronic respiratory conditions including Idiopathic chronic cough [median (SD): 12.8 (3.7)] [16] and Idiopathic pulmonary fibrosis (IPF) [mean (SD): 16.16 (3.66)] [17] indicated a similar degree of cough related morbidity. Although a previous study reported a median LCQ (range): 20.2 (9.5–1.0) [11], this particular study assessed patients with varying degrees of asthma severity, suggesting that our observations are likely to be confined to patients with severe asthma.

The LCQ was initially developed to study idiopathic chronic cough [10]. Existing validated asthma control and quality of life instruments either have no cough-related questions (e.g., ACQ) or limited information on cough related morbidity (e.g., AQLQ -has only one question cough-related question). We identified only modest correlations between LCQ and ACQ, AQLQ, which may suggest that the degree of cough is either cause or consequence of poor disease control and impaired health related quality of life. However further studies evaluating interventions that are known to modify asthma control and quality of life would be required to test whether chronic cough has a causal impact upon asthma control and quality of life in patients with severe asthma. Our observations support the role of LCQ as an additional phenotyping tool in patients with severe asthma.

Gastrointestinal reflux is a common cause of chronic cough and can coincide with asthma, rendering it a potential confounder of cough in asthma. Patients with asthma with self-reported gastrointestinal reflux were predominately female with a significantly lower LCQ compared to patients without self-reported gastrointestinal reflux. In support of this observation previous studies have shown subjects with asthma and concurrent GORD, had poorer asthma control and impaired cough –related quality of life [11]. However, there was no significant difference in LCQ between female patients with asthma, with and without self-reported gastrointestinal reflux. These observations suggest a heightened cough reflex sensitivity that is independent of reflux in female patients with severe asthma which has been well reported previously in female patients with idiopathic chronic cough [18].

One limitation of our study is that we did not quantify gastrointestinal reflux using objective measurements, which is important as reflux is well recognised to be silent in some patients. We also quantified upper airway disease due to rhinitis and nasal polyps using self-reporting, however in our experience self-reported disease demonstrates a high concordance with nasendoscopic findings, suggesting that our observations demonstrating a lack of association of LCQ with upper airways disease are valid.

Cough is also a well-recognised symptom in bronchiectasis and it is important to note that qualitative CT has demonstrated evidence of subclinical bronchiectasis in a proportion of patients (approximately 30%) with severe asthma [19]. Therefore the presence of super added bronchiectasis may have been a potential contributor to cough in this population.

It is believed that chronic cough in asthma may be related to an eosinophilic bronchitis [20]. Airway eosinophils are associated with cough in the presence and absence of airway hyperresponsiveness [20] and mechanistically eosinophils may interact with muscarinic nerves [21] and promote cough reflex hypersensitivity. However, we found no significant relationship between sputum eosinophils (%) and LCQ.

Recent cough guidelines [6] suggest cough reflex hypersensitivity syndrome underlies the aetiology of chronic cough; arising from the hypersensitivity of airway sensory nerves, and thus may be an underlying mechanism attributing to cough in severe asthma. Pharmacological therapies targeting cough reflex hypersensitivity include P2X3 receptor antagonists [22].

## Conclusions

We conclude that a significant proportion of patients with severe asthma have a high degree of cough-related morbidity that appears independent of eosinophilic airway inflammation, is associated with suboptimal disease control and quality of life. Our preliminary findings suggest that further research is now required to validate the LCQ and its responsiveness in severe asthma populations to capture cough-related morbidity.

## Abbreviations

A&E: Accident and emergency
ACQ-6: Six-point juniper asthma control score
AQLQ: Asthma quality of life questionnaire
ATS: American thoracic society
BTS: British thoracic society
COPD: Chronic obstructive pulmonary disease
ERS: European respiratory society
FEV_1_: Forced expiratory volume in one second
FVC: Forced vital capacity
GINA: Global initiative for asthma
GLI: Global lung function initiative
GP: General practice
ICS/LABA: Inhaled corticosteroids/long acting beta-2 agonist combination therapy
LCQ: Leicester cough questionnaire
MCID: Minimal clinically important difference
SD: Standard deviation
